# Editorial perspective: protective factors following cumulative childhood adversity

**DOI:** 10.1192/bjo.2024.31

**Published:** 2024-04-23

**Authors:** Camilla H. Parker, Helen Minnis, Dennis Ougrin

**Affiliations:** East London NHS Foundation Trust, London, UK; and Youth Resilience Unit, Centre for Psychiatry and Mental Health, Wolfson Institute of Population Health, Queen Mary University of London, UK; Institute of Health and Wellbeing, Academic CAMHS, West Glasgow Ambulatory Care Hospital, University of Glasgow, UK; Youth Resilience Unit, Centre for Psychiatry and Mental Health, Wolfson Institute of Population Health, Queen Mary University of London, UK

**Keywords:** Childhood experience, trauma and stressor-related disorders, protective factors, adversity, psychosocial outcomes

## Abstract

Adverse childhood experiences can have a significant impact on adult psychosocial outcomes. However, negative outcomes are not inevitable, and protective factors can interrupt the realisation of negative developmental trajectories and result in positive adaptation in spite of childhood adversity. Interventions that promote social support, encourage education and academic achievement, and address specific personality and dispositional factors are likely to beneficial for those with experience of childhood adversity. Holistic assessment that considers both neurodevelopmental conditions and trauma symptoms is also important for promoting resilience and avoiding assumptions that mental and behavioural problems in children with cumulative adversity are purely ‘social’.

Addressing the health needs of individuals exposed to adverse childhood experiences (ACEs) is a global challenge. ACEs are severe stressful events during childhood including maltreatment (abuse and neglect); serious household dysfunction such as alcohol and substance misuse; and peer, community and collective violence threatening physical and psychological health and development. ACEs can be measured prospectively in childhood or retrospectively in adulthood, although these different measures have been shown to have poor agreement.^1^

We know that experiences of early life adversity are highly prevalent: up to half of all Western children will experience at least one ACE. There are also significant societal costs, with the annual estimated costs attributable to ACEs in European countries equivalent to between 1.1% and 6.0% of GDP.^2^ Evidence exists for a dose-response relationship between ACE exposure and negative psychosocial outcomes in adulthood: the greater the number of ACEs, the greater the risk of mental health problems including neurodevelopmental conditions (NDCs), physical health problems, health-harming behaviours (smoking, physical inactivity, drug and alcohol use) and being a victim or perpetrator of violence.^3,4^ Studies comparing individuals with cumulative ACEs (defined as ≥4 ACEs) to no ACEs found increased risk for attempted suicide (odds ratios (ORs) >12); anxiety and depression (ORs of 3–4); problematic drug use (ORs >10); problematic alcohol use (ORs 5–7); violence victimisation and perpetration (ORs of 7–8); and cancer, cardiovascular and respiratory disease (ORs 2–3).^4,5^ It is important to note that a causal relationship between ACEs and adult psychosocial problems has not been proven, especially with regard to associations between ACEs and NDCs, which may be bidirectional and may underpin much psychopathology.^5^

Given these harmful sequelae, public health interventions focused on primary prevention of ACEs before they occur is essential. There is also substantial multidisciplinary interest in identifying protective factors that might be amenable to intervention and be associated with positive adaptation in spite of adversity. This editorial will introduce the theory underpinning protective factors after cumulative childhood adversity, discuss the evidence base for specific domains of protective factors and consider the potential of translational clinical interventions aiming to harness the power of protective factors in children with experience of adversity.

## Setting the scene: definitions of ACE, psychosocial outcomes and protective factors

Negative outcomes after childhood adversity are not inevitable; individuals who experience ACEs can still flourish later in life despite experiencing significant early adversity. Protective factors operate to interfere with or disrupt the realisation of developmental trajectories between risk factors, such as ACEs, and poor health and social outcomes. Theories of resilience have evolved from being perceived as a stable trait of relative invulnerability to stressors, to that of a dynamic process of interaction between the individual and their environment.^6^ This process is partly influenced by genetic predispositions of temperament and stress physiology, and social factors such as early attachment and social learning. ‘Positive adaptation’ in the face of adversity can be understood as the presence of a positive outcome (e.g. financial security) or the absence of a negative outcome (e.g. absence of psychopathology) after exposure to adversity.

## What protective factors show promise?

The systematic review and narrative synthesis in *BJPsych Open* by Buchanan et al^7^ summarises the current evidence from longitudinal studies of protective factors for adult psychosocial outcomes after cumulative childhood adversity (CA) in multiple domains. Although the authors focused solely on mediators, and protective factors can both be experienced at the same time as childhood adversity and potentially also act as moderators, we focus on their findings as a useful way to consider areas future researchers may wish to focus on. The focus the authors observed was that social support and education were the most common protective factors, followed by certain personality and dispositional factors. These will each be considered individually, in addition to the relevance of neurodevelopmental conditions to childhood adversity.

## Social support

First, the authors found that social support acted as a protective factor for mental health outcomes, particularly depressive symptoms. Benefits were found from both social support quality and quantity individually, with greater effects combined than either on their own. These findings are consistent with the wider literature on social support as a protective factor for mental health in the context of trauma.^8^ The ‘buffering hypothesis’ posits that social support mitigates the negative consequences of stress when perceived availability of interpersonal resources is responsive to the needs elicited by stressful events. The ability to seek and accept comfort and help after stressful events is essential for healthy child and adolescent development. ACEs can disrupt the development of secure attachment relationships and limit access to necessary emotional and physical resources, making social support all the more vital. Social group membership, and the social identities and psychological resources these provide, may be particularly vital.

## Education and academic attainment

Second, Buchanan et al^7^ also found that education and academic attainment were associated with improved outcomes for mental health, socioeconomic status and maltreatment perpetration. This is consistent with prior evidence supporting the protective influences of education, particularly for disadvantaged children, but delineating the unique contribution of specific aspects of education from confounders is complex. A prospective twin cohort study found a history of childhood maltreatment was associated with twice the risk for poor educational and occupational attainment.^9^ Interestingly, the presence of a supportive adult was protective for later occupational outcomes, highlighting the potential positive influence of teachers as mentors and role models. Duration of education is correlated with cognitive abilities with each additional year in education improving IQ by 1–5 points,^10^ with implications for future employment and financial security. Moreover, childhood IQ predicts risk for adult psychopathology which may explain the protective influence of education.

## Personality and dispositional factors

Third, Buchanan et al^7^ found that a variety of personality and dispositional factors were protective for mental health problems in adulthood. These included self-esteem, self-regulation, self-awareness, optimism, mastery and openness to experience. Previous research has largely reported negative cognitive factors, including maladaptive schemas, shame, negative core beliefs and self-criticism as mediating the childhood adversity–psychopathology relationship.^11^ However, these are largely conceptually consistent. Mediation analyses should be interpreted with caution when considering whether these intrapersonal factors are in fact protective factors for mental health. ACEs can exert pervasive influences on personality development, and it is possible such intrapersonal factors may well reflect the response to adversity. Children exposed to adversity may develop mental schema and behaviours to adapt and survive in the context of adversity but which in other contexts are maladaptive and predispose to later psychopathology.

## Neurodevelopmental awareness

An important additional domain relevant to the question of protective factors for cumulative childhood adversity is neurodevelopmental awareness. Children with neurodevelopmental conditions are at higher risk of experiencing severe adversities such as abuse and neglect, but mechanisms and directions of causality are still unclear and may be bidirectional.^5^ For example, behavioural genetic analysis suggests that abuse and neglect does not cause these heritable conditions (that include ADHD, autism and intellectual disability).^12^ Having a child with a neurodevelopmental condition can be stressful for parents and parental stress is an important risk factor for child abuse and neglect. However, there are certain extreme circumstances (e.g. very low stimulation institutional care or severe malnutrition) where severe early adversity does appear to cause neurodevelopmental conditions. Unfortunately, structural inequalities can also play a part in the interplay between neurodevelopmental conditions and adversity: if a child has experienced ACEs then they are at risk of a missed or late diagnosis of a neurodevelopmental condition – this is also true for Black children, who are more likely to have a delayed or missed diagnosis of autism or ADHD compared to White children.^13,14^ An important strategy for increasing resilience in children who have experienced childhood adversity, therefore, is ensuring that children receive a holistic assessment covering both neurodevelopmental and trauma-related symptoms, being careful not to assume that any behavioural problems are ‘social’.^15^

## Translational implications for clinical practice

There is an urgent need for clinicians to be able to offer evidence-based interventions to prevent and treat ACE-related trauma symptoms. A treatment with established evidence in traumatised children and adolescents is trauma-focused cognitive behavioural therapy (TF-CBT). One challenge for clinicians is that children exposed to cumulative childhood adversity often develop severe and complex socioemotional difficulties. Waiting until problems have developed can mean adverse sequelae are more entrenched and difficult to treat. Early years mental health interventions are likely to be more effective due to more pliable infant brain neuroplasticity. We will discuss three novel interventions being evaluated in randomised controlled trials (RCTs).^16–18^ The first is the New Orleans Intervention Model (NIM) for abused and neglected children aged 0–5 years in foster care, which is being compared with social work as usual in the BEst Services Trial (BeST)?^16^ NIM is an infant mental health intervention offering attachment-focused assessment and intensive treatment to children in foster care and their birth families. If timely and adequate change is made, recommendations are made to the court about permanent return to birth families. If not, recommendation is made for adoption. Other interventions currently undergoing RCT evaluation include Dyadic Developmental Psychotherapy (DDP) in the Relationships In Good Hands Trial (RIGHT), intensive attachment-focused psychotherapy for adopted or fostered children aged 5–12 years with a history of maltreatment and relational difficulties;^17^ and the development of an Infant Parent Support (IPS) service for under-5s with a social worker and mental health concerns.^18^

The need to harness protective factors for children with cumulative adversity is gaining political traction. The Scottish government has recently pledged £3.2 million towards infant mental health services, and the Royal College of Psychiatrists has recently published a report ‘Infant and early childhood mental health: the case for action.’ Nothing less will do for this group of patients.
